# Multiple Renal Arteries as a Potential Contributor to Hypertension in Children and Young Adults

**DOI:** 10.3390/jcm15072610

**Published:** 2026-03-29

**Authors:** Ugo Giordano, Benedetta Leonardi, Giulia Cafiero, Marcello Chinali, Alessandro Arena, Flavia Cobianchi Bellisari, Eliana Tranchita, Federica Gentili, Maria Chiara Matteucci, Aurelio Secinaro

**Affiliations:** Bambino Gesù Children’s Hospital, IRCCS, 00146 Rome, Italy; ugo.giordano@opbg.net (U.G.); giulia.cafiero@opbg.net (G.C.); marcello.chinali@opbg.net (M.C.); alessandro.arena@opbg.net (A.A.); flavia.cobianchi@opbg.net (F.C.B.); eliana.tranchita@opbg.net (E.T.); federica.gentili@opbg.net (F.G.); mcmatteucci@gmail.com (M.C.M.); aurelio.secinaro@opbg.net (A.S.)

**Keywords:** arterial hypertension, renal vascular variants, pediatric hypertension, left ventricular hypertrophy, CT angiography

## Abstract

**Background:** Arterial hypertension in childhood is an increasing health concern, often associated with structural and functional cardiovascular or renal alterations. This study aimed to investigate the prevalence and type of non-stenotic renal artery anatomical variants in children with systemic hypertension and to assess their possible association with cardiac involvement. **Methods**: A total of 107 children and adolescents with hypertension (mean age 15.4 ± 2.7 years) were evaluated. Hypertension was defined as blood pressure persistently above the 95th percentile for over one year, confirmed by 24 h ambulatory blood pressure monitoring. Patients with known secondary causes were excluded. All underwent renal vascular imaging by CT or MRI and echocardiographic assessment of left ventricular morphology and function. **Results**: Renal artery anatomical variants were found in 69 of 107 patients (65%), mainly unilateral or bilateral accessory polar arteries. Other anomalies found (left renal vein narrowing or duplication, severe left renal artery stenosis) were excluded from the statistical analysis. Normal renal vasculature was observed in only 32%. Left ventricular hypertrophy was detected in 41%, highlighting a significant prevalence of target-organ involvement. No statistically significant differences were found in terms of hypertension or hypertrophy between patients with renal artery anatomical variants and those without. However, patients with renal anomalies more frequently required dual antihypertensive therapy (*p* = 0.025). **Conclusions**: Renal artery anatomical variants, even in the absence of overt stenosis, may contribute to the pathogenesis of pediatric hypertension and complicate its management. Systematic evaluation of renal vasculature should be considered in the diagnostic workup to improve risk stratification and guide management strategies.

## 1. Introduction

Arterial hypertension (AH) is a major global health problem, responsible for approximately 12.8% of all deaths, including more than half of stroke-related deaths and nearly half of deaths from coronary heart disease [1,2,3,4,5,6]. Although traditionally considered a disease of adulthood, its prevalence in children and adolescents has been steadily increasing over the past decades. In the United States, about one third of the adult population is hypertensive [5], and the upward trend in childhood blood pressure has been well documented. Data from the National Health and Nutrition Examination Survey (NHANES) show a shift in the entire blood pressure distribution in youth, with systolic and diastolic pressures rising by 1.4 and 3.3 mmHg, respectively, from 1963 to 2002 [5]. Much of this increase has been attributed to the obesity epidemic, which strongly correlates with higher rates of both pre-hypertension and sustained hypertension in the pediatric population.

Hypertension in childhood is clinically relevant because it not only predicts adult hypertension but is also associated with early signs of target organ damage. Studies have demonstrated that hypertensive children may already show increased left ventricular mass and geometric remodeling of the heart, while even adolescents with pre-hypertension have higher left ventricular mass than their normotensive peers [7,8,9].

Pediatric hypertension is classified as primary when no underlying cause can be identified, and secondary when hypertension arises from conditions such as renal parenchymal disease, coarctation of the aorta, endocrine disorders, or genetic syndromes. While secondary hypertension was once more frequent in children, primary hypertension now accounts for the majority of cases, particularly in adolescents [2,3,10,11,12]. However, emerging evidence suggests that vascular anomalies of the renal circulation—such as accessory renal arteries or variations in venous drainage—might play an under-recognized role in the development of hypertension [13,14,15].

In adults, accessory renal arteries have been proposed as a possible cause of so-called primary hypertension, likely through hemodynamic mechanisms that reduce renal segmental perfusion and activate the renin–angiotensin system. However, data in pediatric and young adult populations remain limited. To our knowledge, no systematic investigation has evaluated the prevalence and clinical significance of non-stenotic renal artery anatomical variants in hypertensive children. The present study was therefore designed with two primary objectives: (1) to determine the prevalence of renal artery anatomical variants in children and young adults with apparently primary hypertension, and (2) to assess whether the presence of such anomalies is associated with an increased prevalence of cardiac target-organ damage compared with patients exhibiting normal renal vasculature.

## 2. Materials and Methods

### 2.1. Study Design and Population

This single-center observational study was conducted at the Bambino Gesù Children’s Hospital, which has operated a dedicated outpatient clinic for pediatric blood pressure assessment since 2005. The study is based on data collected from patients with arterial hypertension who were evaluated and followed at our institution between 2005 and 2025.

Eligible participants included children, adolescents, and young adults aged 6–25 years, referred either by their primary care physicians following the detection of elevated blood pressure during routine examinations or by other hospital departments. Patients were eligible if they had confirmed persistent hypertension, defined as repeated office blood pressure (BP) measurements above the 95th percentile for age, sex, and height despite a 3-month low-sodium dietary intervention, and confirmed by 24 h ambulatory blood pressure monitoring (ABPM). Only normal-weight patients were included in order to minimize the confounding effect of obesity. Exclusion criteria comprised all known causes of secondary hypertension, including renal artery stenosis, renal parenchymal disease, coarctation of the aorta, endocrine disorders, diabetes mellitus, genetic syndromes (e.g., Williams, neurofibromatosis), chronic drug use (steroids), systemic infections, organ dysfunction, or recent surgery. Including adult women in the study population, we excluded those receiving estrogen therapy in order to avoid potential confounding effects on blood pressure regulation and renin–angiotensin–aldosterone system activity.

### 2.2. Blood Pressure Measurement and ABPM

All patients underwent 24 h ambulatory blood pressure monitoring (ABPM) (Spacelab 90207^®^, Spacelab Inc., Redmond, WA, USA). The appropriate cuff size was chosen with the same criteria used at rest. The subject was instructed to relax and extend the arm during blood pressure measurements. Measurements that produced a pulse pressure of less than 20 mmHg or a heart rate lower than 40 beats per minute were regarded as errors and excluded automatically by the recorder. If any of the parameters could not be measured correctly, the measurement was repeated within 3 min. The device was set to obtain readings every 15 min from 7:00 a.m. to 11 p.m. and every 30 min from 11:00 p.m. to 7:00 a.m. Awake and sleep periods were established by the daily-activities diary kept by each patient. Ambulatory blood pressure readings were only accepted when at least 75% of the measurements were successful. The mean of 24 h daytime and night-time systolic and diastolic blood pressures, 24 h mean arterial pressure and presence of the physiological nocturnal dipping were determined. To define hypertension, references values obtained from the oscillometric mean ABPM values in healthy children table adjusted for sex and height were used [16,17]. The percentage of nighttime reduction in BP (‘dipping’) was calculated as (daytime BP − nighttime BP) × 100/daytime BP. Non-dipping was defined as a nocturnal BP reduction of <10%. Antihypertensive therapy was evaluated, and it was recorded whether patients were receiving single-drug therapy or dual antihypertensive therapy.

### 2.3. Laboratory Tests

Blood samples were obtained to exclude secondary causes of hypertension, including assessments of glucose and lipid profile, renal function (glomerular filtration rate, renin), adrenal function (aldosterone, cortisol, catecholamines, metanephrines, chromogranin), pituitary function (ACTH), and thyroid function. In addition, all patients underwent urine dipstick testing and measurement of 24 h urinary protein or albumin excretion. All laboratory tests were performed at baseline, prior to the initiation of antihypertensive therapy.

### 2.4. Imaging Evaluation

Doppler renal ultrasound was performed in all patients as the first-line screening modality for renal artery disease. A renovascular etiology of hypertension was considered when stenosis was directly visualized, a parvus et tardus waveform was observed, or age-specific pathological flow parameters were detected.

All patients underwent either abdominal computed tomography (CT) angiography or contrast-enhanced magnetic resonance angiography (MRI).

The choice of imaging modality reflected the evolution of our institutional protocol over time. Initially, magnetic resonance angiography (MRI) was preferentially used in order to minimize radiation exposure in accordance with the ALARA (As Low As Reasonably Achievable) principle. However, as our experience increased, limitations of MRI became evident, particularly in the evaluation of small-caliber vessels and accessory renal arteries in younger children. For this reason, CT was progressively adopted in selected cases, as it provides higher spatial resolution, faster acquisition times, and more reliable visualization of small accessory arteries and complex vascular anatomy.

Abdominal CT angiography was performed with a second-generation dual-source CT system (Definition Flash, Siemens Healthcare, Forchheim, Germany), equipped with two X-ray tubes and two detectors positioned at a 95° angular offset. Image acquisition was conducted using a high-pitch spiral technique (“Flash Spiral”). Scan parameters were as follows: pitch 1.55, collimation 96 × 0.6 mm, gantry rotation time 0.25 s, reference mAs (tube current modulation, CARE Dose) adjusted to body weight (60–100 mAs), 90 kV, and total scan duration 1.24 s.

All examinations included an arterial phase obtained during the injection of a low-osmolar non-ionic iodinated contrast agent at high flow rate. Contrast volume was tailored to patient body size and administered via a peripheral vein with a dual-syringe power injector. Image acquisition was initiated manually using bolus tracking in the ascending aorta. Post-processing included multiplanar reformations (MPRs), three-dimensional volume-rendered reconstructions (3D-VR), and maximum-intensity projections (MIPs) for comprehensive data evaluation.

MR imaging was performed on a 1.5 Tesla scanner (MAGNETOM Aera or Sola, Siemens Healthineers, Erlangen, Germany). The imaging protocol consisted of axial and coronal T1- and T2-weighted sequences, as well as 3D contrast enhancement MRI for renal vascular assessment. A gadolinium-based contrast agent (0.2 mmol/kg) was administered using an automatic injector. Following acquisition of raw data, MR angiograms were reconstructed with the maximum-intensity projection algorithm.

Renal arteries were classified as normal or stenotic. Accessory arteries were defined according to the Merklin and Michels classification, based on their origin (abdominal aorta, main renal artery, or other vessels) and point of entry (upper polar, hilar, or lower polar).

### 2.5. Echocardiography

Comprehensive transthoracic echocardiography was performed using standardized methods. Left ventricular dimensions and wall thicknesses were measured, and left ventricular mass (LVM) was indexed to height according to the allometric relation LVM/height^2.16^ + 0.09 in patients up to 18 years of age [18]. In patients older than 18 years, LVM was indexed to height^2.7^ [19]. Left ventricular hypertrophy (LVH) was defined as an LVM index ≥ 45 g/m^2.16^ + 0.09 for patients up to 18 years of age or as an LVM index ≥ 50 g/m^2.7^. Relative wall thickness was calculated to assess left ventricular concentric geometry using age specific reference values [20].

### 2.6. Statistical Analysis

Descriptive statistics are presented as median with interquartile range (IQR) for continuous variables and as counts and percentages for categorical variables. Normality of continuous variables was assessed using the Shapiro–Wilk test; accordingly, the median was used as a measure of central tendency. For the comparison of continuous variables that were not normally distributed, the Wilcoxon rank sum test (for two groups) was applied. Categorical variables were compared using the Pearson chi-squared test. A power analysis was performed using G*Power software (Version 3.1) to estimate the minimum sample size required to achieve a statistical power (1 − β) of at least 0.80 for the comparison between patients with and without renal artery anatomical variants. Because the main comparisons involved non-parametric tests, the analysis was based on the Wilcoxon rank sum test using the Asymptotic Relative Efficiency (ARE) method. A small effect size (Cohen’s d = 0.2) was assumed, with a two-tailed α significance level of 0.05 and an allocation ratio of 1:1 between groups. Under these assumptions, the estimated sample size required to achieve a power of 0.80 was 912 subjects (456 per group).

## 3. Results

Over a 5-year period, 874 children were evaluated for suspected hypertension. Of these, 107 patients (12%) met the inclusion criteria and completed the comprehensive diagnostic protocol, which included 24-h ambulatory blood pressure monitoring (ABPM), blood analyses, and either CT or MRI to assess for renal artery anomalies.

Renal artery anatomical variants were present in 69 of 107 hypertensive patients (65%), most commonly unilateral or bilateral accessory polar arteries. Among vascular anomalies, double left renal artery (RAR L) was identified in 26 patients, while double right renal artery (RAR R) was present in 24 patients, double right and double left renal arteries (RAR R and RAR L) were found in 17 patients. Less frequent arterial variants included Triple right renal artery and double left renal artery (TAR R and RAR L) in 1 patient and double right renal artery and triple left renal artery (RAR R and TAR L) in 1 patient.

Other vascular anomalies were identified in a total of four patients, including left renal vein narrowing or duplication, as well as one case of severe left renal artery stenosis; these patients were excluded from the analysis. Normal renal vascular anatomy was documented in 34 patients (32%). All patients had persistent hypertension on ABPM. Seventy-four patients (69%) showed elevated daytime systolic BP, 49 (46%) had elevated nighttime systolic BP, 32 (30%) had both, and 15 (14%) had isolated diastolic hypertension. LVH was present in 44 patients (41%). Abnormal left ventricular mass was observed in 14 of 34 patients without renal artery anatomical variants (42%) and in 26 of 69 patients with renal artery anatomical variants (41%), with no significant difference between groups (*p* = 0.865).

Comparisons between hypertensive patients with and without renal artery anatomical variants showed no statistically significant differences in sex distribution, age, ABPM parameters, or left ventricular mass (Table 1), nor in the laboratory findings reported in Table 2. No significant differences in left ventricular mass or ABPM parameters were observed between patients with and without renal artery anomalies, even when analyses were stratified by sex (Table 3). Antihypertensive therapy was evaluated according to the presence of renal artery anomalies. Among patients with renal artery anatomical variants (right, left, bilateral, or accessory; *n* = 69), 47 were receiving antihypertensive treatment (70%), whereas 27 of 34 patients without renal artery anatomical variants (87%) were receiving antihypertensive therapy, with no statistically significant difference between groups (*p* = 0.070). Interestingly, dual therapy was significantly more common in patients with renal artery anomalies, being required in 12 of 47 treated patients (26%) compared with 1 of 27 patients (3.8%) without anomalies (*p* = 0.025) (Table 1). In patients with renal artery anomalies, the most frequently prescribed drug was candesartan (34 patients), followed by amlodipine and olmesartan, while lisinopril, losartan, and moduretic were each used in one patient. The most common second-line agents included carvedilol, amlodipine, ramipril, and doxazosin, while bisoprolol was used in one case. Among the 27 patients without renal artery anatomical variants requiring antihypertensive therapy, candesartan was also the most frequently prescribed drug (25 patients), while amlodipine was used in one patient. The only patient requiring dual therapy (3.8%) received doxazosin as the second agent.

### Renal Vascular Anatomy

**Figure 1:**
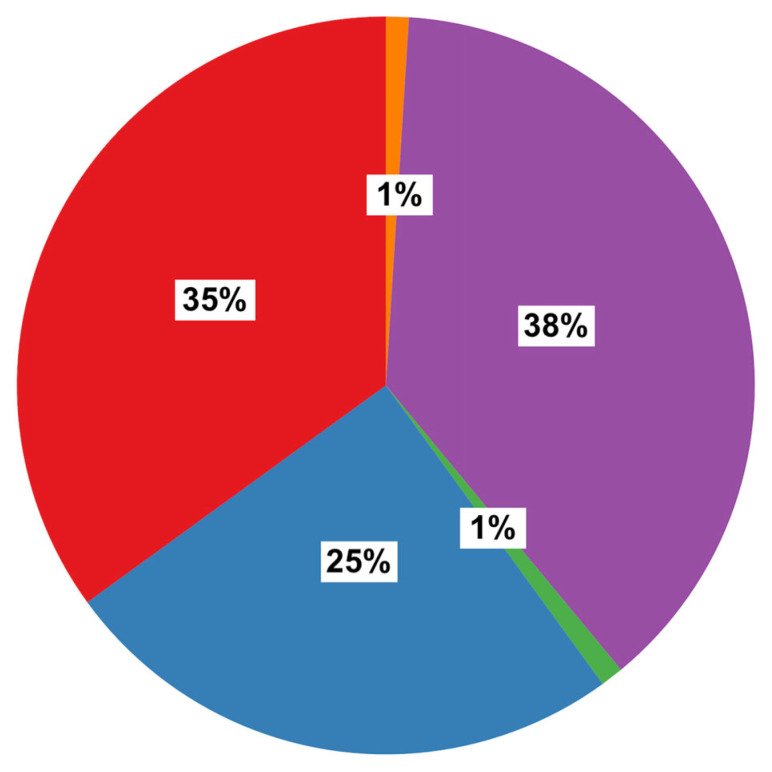
Prevalence of individual renal artery anatomical variants in our cohort. Legend: RAR R (red) = Double right renal artery; RAR R and RAR L (blue) = Double right renal artery and double left renal artery; RAR R and TAR L (green) = Double right renal artery and triple left renal artery; RAR L (purple) = Double left renal artery; TAR R and RAR L (orange) = Triple right renal artery and double left renal artery.

**Figure 2:**
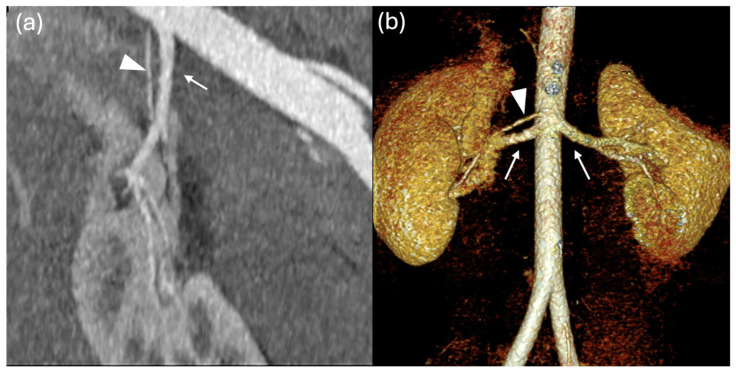
Coronal Maximum Image Projection reconstruction (MIPr) (**a**) and volume rendering (**b**) CT images show the main renal arteries (arrows) and an accessory artery (arrowhead) directed to the inferior pole of the right kidney.

**Figure 3:**
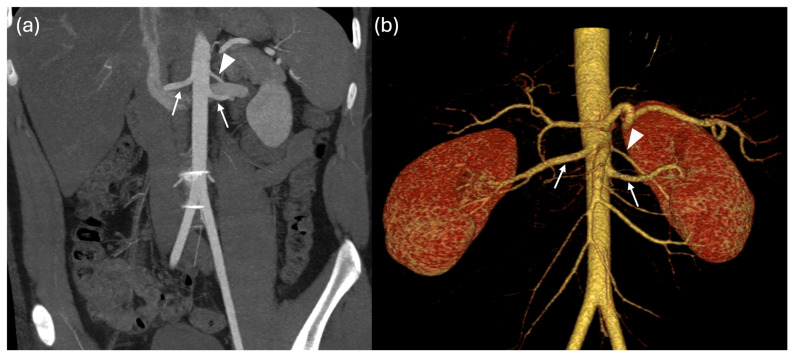
Coronal MIPr (**a**) and volume rendering (**b**) CT images show the main renal arteries (arrows) and an accessory artery (arrowhead) directed to the posterior segments of the left kidney.

**Figure 4:**
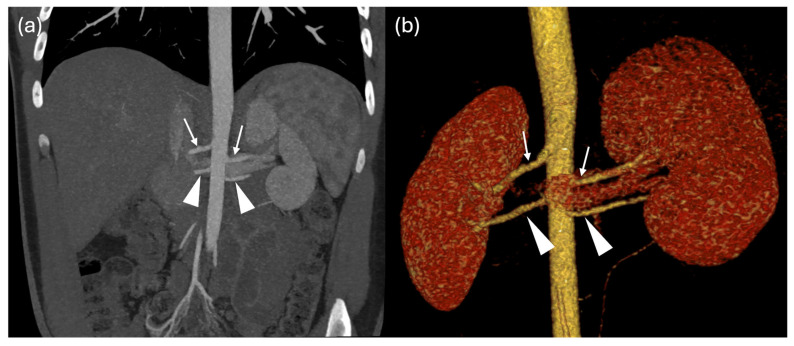
Coronal MIPr (**a**) and volume rendering (**b**) CT images show the main renal arteries (arrows) and a bilateral accessory artery (arrowhead) directed to the inferior pole of both kidneys.

**Figure 5:**
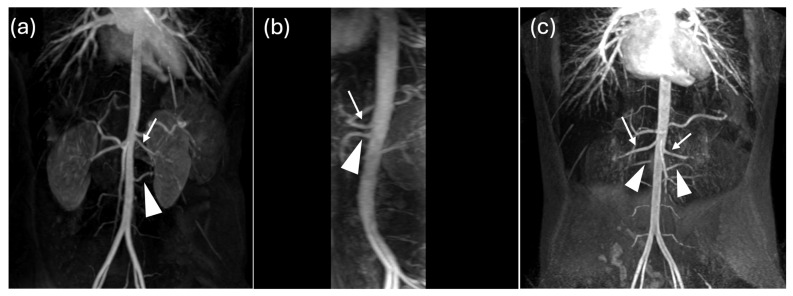
MIPr of Contrast Enhancement Magnetic Resonance Angiography. MR images show the main renal arteries (arrows) and an accessory artery (arrowhead) directed to the left (**a**), right (**b**) and both (**c**) kidneys.

Using both cardiac magnetic resonance and CT imaging, renal artery anatomical variants were identified in 69 out of 107 patients (65%). Twenty-six patients had a RAR L, 24 had a RAR R, 17 had RAR R and RAR L, one patient presented with a TAR R and RAR and another with a RAR R and TAR L (Figure 1, Figure 2, Figure 3, Figure 4 and Figure 5).

In addition, two patients (2%) were found to have renal artery stenosis, one patient had an abrupt caliber reduction in the left renal vein in the interaortomesenteric segment with associated left renal vein ectasia, one had a double left renal vein. Overall, 32 patients (%) demonstrated a completely normal renal vascular pattern.

## 4. Discussion

This study demonstrates a remarkably high prevalence of renovascular anomalies in normal-weight children and young adults with persistent hypertension who would otherwise have been classified as having primary hypertension. The consistent detection of renovascular anomalies in our cohort highlights the importance of considering this possibility when evaluating young individuals with arterial hypertension, even when current therapeutic approaches remain largely limited to pharmacological management.

Given their high prevalence, renal artery anatomical variants may plausibly contribute to the development of secondary (renal) hypertension. Furthermore, hypertension associated with these anomalies may be more difficult to control pharmacologically than primary hypertension. In our study, dual antihypertensive therapy was significantly more frequent among patients with renal artery anomalies, supporting the hypothesis that renal artery anatomical variants may provide a persistent hypertensive stimulus and consequently require more intensive pharmacological management.

Renovascular alterations are known to influence blood pressure regulation through mechanisms such as activation of the renin–angiotensin–aldosterone system (RAAS) and impaired renal perfusion, both of which may contribute to sustained or treatment-resistant hypertension. Regarding renin–angiotensin system activation, plasma renin and aldosterone levels, assessed at baseline prior to the initiation of antihypertensive therapy, were higher in patients with renal artery abnormalities compared to those without, although the differences did not reach statistical significance, suggesting a possible involvement of RAAS activation in this subgroup. In this context, the higher prevalence of dual therapy observed in our cohort may reflect the presence of a renal-driven component in the pathophysiology of hypertension in these patients, necessitating a more aggressive therapeutic strategy to achieve adequate blood pressure control. Therefore, the assessment of renal artery anatomy may represent a clinically relevant element in the evaluation of young hypertensive patients. The clinical significance of accessory renal arteries has been a subject of debate for many decades. Early investigations by Marshall (1951) [14] and later by Geyer (1962) [13] suggested that the prevalence of multiple renal arteries was higher in hypertensive patients compared with normotensive controls, raising the hypothesis that these vascular variants might contribute to the development or maintenance of elevated blood pressure. The proposed mechanisms included alterations in renal blood flow, asymmetric perfusion of the renal parenchyma, and potential effects on the renin–angiotensin–aldosterone system. Although these early observations stimulated considerable interest, subsequent studies have reported conflicting results [21,22], with some confirming a possible association while others failed to demonstrate any significant link [23].

Gupta (2004) [21], using MR angiography, found no significant difference in renal artery stenosis between patients with and without accessory renal arteries, suggesting that these vessels are mainly anatomical variants without clinical impact. Similarly, Liang et al. (2025) [22], in a large retrospective cohort of 1113 patients with primary hypertension, reported no differences in blood pressure control, renal function, echocardiographic parameters, or renin–aldosterone levels, further supporting the notion that accessory renal arteries are unlikely to play a major role in hypertension.

In contrast, a more recent study [23] demonstrated a higher prevalence of congenital renal artery anatomical variants in patients with resistant hypertension compared with well-controlled cases (38.3% vs. 11.7%), with an almost five-fold increased risk. Although this analysis encompassed a broader spectrum of vascular variants, it raises the possibility that such anomalies may contribute to refractory hypertension in selected patients.

The pathophysiological rationale is grounded in hemodynamic principles. According to Poiseuille’s law, vascular resistance increases with vessel length and decreases with the fourth power of the vessel radius. Accessory renal arteries are often longer and of smaller caliber, potentially leading to relative hypoperfusion of the renal parenchyma they supply. This condition may trigger activation of the renin–angiotensin–aldosterone system (RAAS), thereby contributing to increased systemic blood pressure. Although our study did not include direct measurements of renal perfusion, we documented a significant prevalence of renal artery anatomical variants in a pediatric population with persistent hypertension. Furthermore, our data on antihypertensive therapy suggest that these patients may require more intensive pharmacological management and potentially closer longitudinal follow-up.

In our study, the presence of renal artery anatomical variants was not associated with a higher prevalence of increased left ventricular mass compared with patients with primary hypertension. This finding may be explained by the fact that left ventricular hypertrophy is primarily a consequence of elevated blood pressure itself rather than of the underlying cause of hypertension. Nevertheless, our results highlight that hypertension can already lead to significant cardiac remodeling in childhood. In our cohort, more than 40% of patients showed evidence of left ventricular hypertrophy, emphasizing the importance of systematic echocardiographic evaluation in children with hypertension. Although renal artery anatomical variants were not associated with a higher prevalence of increased ventricular mass, the frequent coexistence of these anomalies and left ventricular hypertrophy in our population suggests that clinically relevant cardiovascular effects may already be present in a substantial subset of patients. This observation is particularly concerning given the well-established evidence that early left ventricular hypertrophy represents a major risk factor for adverse cardiovascular outcomes, including diastolic dysfunction, arrhythmias, and progression to heart failure [1,2,3,23,24].

Taken together, our findings extend these observations to the pediatric setting, underscoring the importance of moving beyond renal ultrasound and echocardiography in patients with hypertension that requires combination therapy for adequate control—particularly when target organ damage, such as left ventricular hypertrophy, is already present. In such cases, second-level imaging, such as computed tomography, should be considered to assess the possible presence of renal artery anatomical variants.

The evaluation of renal artery anatomical variants requires imaging techniques with high spatial resolution and multiplanar reconstruction capabilities. Based on our experience with both magnetic resonance imaging and CT in pediatric and young adult patients, CT remains the reference modality for anatomical characterization of the renal vasculature, as it provides high-definition visualization of the main arteries, accessory vessels, segmental branches, and vascular malformations, including supernumerary arteries, early bifurcations, aneurysms, and stenoses. Magnetic resonance angiography (MRA), although offering lower spatial resolution, has the advantage of avoiding exposure to ionizing radiation and iodinated contrast agents and therefore represents a useful alternative in a selected subgroup of pediatric patients, particularly those with impaired renal function.

Larger population-based studies are needed to clarify whether renal artery anatomical variants directly contribute to persistent hypertension in children or whether they act synergistically with other risk factors to accelerate target-organ damage.

Limitations: This study has several limitations. The choice between MRI and CT was not based on specific clinical indications but rather on availability, which may have introduced heterogeneity. The analysis was limited to a single-center cohort with a relatively small sample size, and no normotensive control group was included for comparison. Data on the time course of hypertension were not available. Owing to the retrospective design of the study, reliable and homogeneous information on the duration of hypertension and its longitudinal progression—particularly from the time of first evaluation at our center, including escalation to combination therapy—could not be consistently retrieved. For this reason, this parameter was not included in the analysis and should be considered a limitation of the study, now acknowledged in the revised manuscript.

Although aldosterone and renin levels were higher in patients with renal artery abnormalities, these differences did not reach statistical significance, likely due to the relatively high variability of these parameters and the limited sample size, thus precluding definitive conclusions regarding a possible activation of the renin–angiotensin system in patients with renal artery anatomical variants.

In addition, functional assessments of renal perfusion were not performed, restricting the ability to draw mechanistic conclusions regarding the hemodynamic significance of the vascular anomalies observed.

## 5. Conclusions

Among children evaluated for hypertension that was difficult to control with monotherapy, we identified a substantial proportion with renal artery anatomical variants. These findings suggest that the presence of a duplicated renal artery, even in the absence of significant stenosis, may contribute to the development and persistence of pediatric hypertension and may require more intensive antihypertensive management. Therefore, in selected pediatric and young adult patients with persistent hypertension, a more comprehensive vascular assessment beyond renal artery ultrasound alone may be warranted.

## Figures and Tables

**Table 1:** Ambulatory blood pressure data and left ventricular mass in hypertensive patients with and without renal artery anomalies.

Characteristic	Overall N = 103 ^1^	Normal N = 34 ^1^	Renal Artery Anatomical Variants N = 69 ^1^	*p*-Value ^2^
Gender (M)	84 (82%)	29 (85%)	55 (80%)	0.492
Age (years)	15.8 (13.0, 17.7)	16.2 (13.8, 17.7)	15.5 (12.6, 17.5)	0.360
bmi (kg/m^2^)	22.8 (20.4, 24.8)	22.4 (20.3, 24.7)	23.0 (20.9, 24.9)	0.766
Patients taking antihypertensive therapy (n, %)	74 (76%)	27 (87%)	47 (70%)	0.070
Dual therapy (n, %)	13 (18%)	1 (3.8%)	12 (26%)	**0.025**
LVM	43 (36, 50)	44 (37, 55)	43 (36, 49)	0.623
24 h mean SBP (mmHg)	127 (120, 134)	129 (123, 135)	127 (120, 133)	0.394
Daytime SBP 06–23 (mmHg)	132 (124, 138)	135 (126, 140)	131 (123, 138)	0.233
Nighttime SBP 23–06 (mmHg)	119 (111, 125)	121 (112, 126)	118 (111, 125)	0.634
24 h mean DBP (mmHg)	70 (64, 75)	70 (66, 77)	69 (63, 74)	0.226
Daytime DBP 06–23 (mmHg)	73 (67, 80)	75 (71, 81)	73 (66, 80)	0.172
SBP dip (%)	10 (7.0, 13.0)	10.9 (8.1,13.7)	9.7 (6.2, 12.9)	0.207
Nighttime DBP 23–06 (mmHg)	59 (55, 66)	59 (54, 67)	59 (55, 64)	0.960

Legend: LVM = left ventricular mass index, indexed as g/m^2.7^ in adults and g/m^2.16^ in pediatric patients; 24 h mean SBP = 24 h mean systolic blood pressure; Daytime SBP (06–23 h) = mean systolic blood pressure during daytime hours; Nighttime SBP (23–06 h) = mean systolic blood pressure during nighttime hours; 24 h mean DBP = 24 h mean diastolic blood pressure; Daytime DBP (06–23 h) = mean diastolic blood pressure during daytime hours; Nighttime DBP (23–06 h) = mean diastolic blood pressure during nighttime hours; SBP dip = systolic blood pressure dip. ^1^ n (%); Median (Q1, Q3); ^2^ Pearson’s Chi-squared test; Wilcoxon rank sum test.

**Table 2:** Laboratory parameters according to renal artery abnormalities.

Parameter	Normal (n = 34)	Renal Artery Abnormalities (n = 69)	*p*-Value
Plasma aldosterone (upright), ng/dL	106.9 ± 38.6 (105.5) [22.7–160.0]	130.4 ± 77.3 (120.0) [5.2–304.0]	0.528
Plasma renin (upright), µIU/mL	24.9 ± 12.9 (26.0) [2.0–46.0]	44.3 ± 37.0 (32.8) [8.3–163.0]	0.115
Blood urea nitrogen, mg/dL	14.7 ± 3.3 (14.0) [10–21]	14.4 ± 3.3 (14.0) [8–22]	0.842
Creatinine, mg/dL	0.81 ± 0.15 (0.83) [0.50–1.18]	0.74 ± 0.21 (0.72) [0.36–1.32]	0.153
TSH, mIU/L	2.65 ± 1.58 (2.16) [0.96–6.17]	2.39 ± 1.27 (2.11) [0.75–5.89]	0.605
FT4, ng/dL	1.29 ± 0.16 (1.24) [1.05–1.53]	1.32 ± 0.28 (1.29) [0.95–2.47]	1.000
Chloride, mmol/L	102.6 ± 2.2 (102) [98–108]	103.9 ± 6.8 (103) [90–141]	0.476
Potassium, mmol/L	4.35 ± 0.28 (4.37) [3.89–4.83]	4.37 ± 0.38 (4.39) [3.50–4.96]	0.669
Sodium, mmol/L	139.5 ± 1.5 (139) [137–142]	140.4 ± 1.7 (140) [138–144]	0.120

Legenda: Values are expressed as mean ± standard deviation (median) [min–max]. Group comparisons were performed using the Wilcoxon rank sum test. No abnormalities = patients without renal artery anatomical variants; Renal artery abnormalities = patients with renal artery anatomical variants.

**Table 3:** Ambulatory blood pressure data and left ventricular mass stratified by sex.

	Males				Females			
Characteristic	Overall	Normal	Renal Artery Anatomical Variants	*p*-Value ^2^	Overall	Normal	Renal Artery Anatomical Variants	*p*-Value ^3^
	N = 84 ^1^	N = 29 ^1^	N = 55 ^1^		N = 19 ^1^	N = 5 ^1^	N = 14 ^1^	
Age (Years)	16.1 (14.2, 17.7)	16.2 (14.3, 17.7)	16.0 (14.2, 17.7)	0.873	12.9 (10.0, 15.5)	14.0 (13.1, 16.4)	12.5 (9.8, 13.2)	0.107
bmi (kg/m^2^)	23.1 (20.9, 25.4)	22.4 (20.3, 24.7)	23.2 (21.8, 25.9)	0.354	21.8 (18.7, 24.0)	22.4 (21.3, 24.7)	21.7 (18.7, 22.3)	0.343
LVM	44 (37, 53)	43 (36, 55)	44 (37, 50)	0.921	38 (32, 48)	44 (41, 47)	36 (32, 48)	0.687
LVH (%)	43 (55%)	15 (54%)	28 (56%)	0.836	13 (68%)	3 (60%)	10 (71%)	>0.999
24 h mean SBP (mmHg)	128 (121, 135)	130 (123, 136)	127 (121, 133)	0.579	123 (116, 132)	124 (124, 132)	122 (116, 131)	0.378
Daytime SBP 06–23 (mmHg)	132 (124, 139)	136 (128, 141)	132 (124, 138)	0.210	126 (122, 136)	126 (126, 135)	126 (122, 139)	0.963
Nighttime SBP 23–6 (mmHg)	119 (111, 126)	118 (111, 126)	119 (112, 126)	0.826	117 (110, 122)	122 (120, 124)	114 (108, 120)	0.095
24 h mean DBP (mmHg)	69 (64, 74)	70 (64, 75)	68 (63, 73)	0.286	70 (64, 79)	70 (70, 79)	70 (62, 78)	0.486
Daytime DBP 06–23 (mmHg)	73 (66, 80)	75 (71, 80)	72 (66, 79)	0.195	74 (68, 85)	75 (73, 84)	74 (67, 85)	0.610
Nighttime DBP 23–6 (mmHg)	59 (55, 64)	59 (54, 67)	59 (55, 63)	0.815	63 (57, 72)	62 (62, 73)	63 (57, 70)	0.676
SBP dip (%)	10.2 (7.0, 13.6)	11 (8.4, 14.2)	9.6 (6.1, 13.0)	0.098	9.6 (6.9–10.3)	7.5 (6.9–10.3)	9.7 (9.4–10.9)	0.079

Legend: LVM = left ventricular mass index, indexed as g/m^2.7^ in adults and g/m^2.16^ in pediatric patients; Daytime SBP (06–23 h) = mean systolic blood pressure during daytime hours; Nighttime SBP (23–06 h) = mean systolic blood pressure during nighttime hours; 24 h mean DBP = 24 h mean diastolic blood pressure; Daytime DBP (06–23 h) = mean diastolic blood pressure during daytime hours; Nighttime DBP (23–06 h) = mean diastolic blood pressure during nighttime hours; SBP dip = systolic blood pressure dip. ^1^ Median (Q1, Q3); ^2^ Wilcoxon rank sum test; ^3^ Wilcoxon rank sum exact test; Wilcoxon rank sum test.

## Data Availability

The datasets generated and analyzed during the current study are not publicly available due to institutional privacy policies and ethical restrictions of our hospital.

## Abbreviations

The following abbreviations are used in this manuscript:

CT	Computed Tomography
MRI	Magnetic Resonance Angiography
AH	Arterial Hypertension
ABPM	Ambulatory Blood Pressure Monitoring
BP	Blood pressure
MPRs	Multiplanar Reformations
MIPs	Maximum-Intensity Projections
LVM	Left Ventricular Mass
LVH	Left Ventricular Hypertrophy
